# Comprehensive chronic lymphocytic leukemia diagnostics by combined multiplex ligation dependent probe amplification (MLPA) and interphase fluorescence in situ hybridization (iFISH)

**DOI:** 10.1186/s13039-014-0079-2

**Published:** 2014-11-19

**Authors:** Eyad Alhourani, Martina Rincic, Moneeb AK Othman, Beate Pohle, Cordula Schlie, Anita Glaser, Thomas Liehr

**Affiliations:** Jena University Hospital, Friedrich Schiller University, Institute of Human Genetics, Kollegiengasse 10, D-07743 Jena, Germany; Croatian Institute of Brain Research, Salata 12, 1000 Zagreb, Croatia

**Keywords:** Chronic lymphocytic leukemia (CLL), Chromosomal aberrations, Multiplex ligation-dependent probe amplification (MLPA), Fluorescence in situ hybridization (FISH)

## Abstract

**Background:**

Banding-karyotyping and metaphase-directed-fluorescence-in-situhybridization (FISH) may be hampered by low mitotic index in leukemia. Interphase FISH (iFISH) is a way out here, however, testing many probes at the same time is protracted and expensive. Here multiplex-ligation-dependent-probe-amplification (MLPA) was used retrospectively in chronic lymphocytic leukemia (CLL) samples initially studied by banding cytogenetics and iFISH. Detection rates of iFISH and MLPA were compared and thus a cost-efficient scheme for routine diagnostics is proposed.

**Results:**

Banding cytogenetics was done successfully in 67/85 samples. DNA was extracted from all 85 CLL samples. A commercially available MLPA probe set directed against 37 loci prone to be affected in hematological malignancies was applied. Besides, routine iFISH was done by commercially available probes for following regions: 11q22.3, 12p11.2-q11.1, 13q14.3, 13q34, 14q32.33 and 17p13.1. MLPA results were substantiated by iFISH using corresponding locus-specific probes.

Aberrations were detected in 67 of 85 samples (~79%) applying banding cytogenetics, iFISH and MLPA. A maximum of 8 aberrations was detected per sample; however, one aberration per sample was found most frequently. Overall 163 aberrations were identified. 15 of those (~9%) were exclusively detected by banding cytogenetics, 95 were found by MLPA (~58%) and 100 (~61%) by routine iFISH. MLPA was not able to distinguish reliably between mono- and biallelic del(13)(q14.3q14.3), which could be easily identified as well as quantified by routine iFISH. Also iFISH was superior to MLPA in samples with low tumor cell load. On the other hand MLPA detected additional aberrations in 22 samples, two of them being without any findings after routine iFISH.

**Conclusions:**

Both MLPA and routine iFISH have comparable detection rates for aberrations being typically present in CLL. As MLPA can detect also rare chromosomal aberrations it should be used as an initial test if routine cytogenetics is not possible or non-informative. Still iFISH should be used additionally to distinguish mono- from biallelic deletions and also to determine rate of mosaicism for 13q14.2 to 13q14.3. In case MLPA is negative the corresponding CLL samples should be tested at least by iFISH using the standard probe set to.

**Electronic supplementary material:**

The online version of this article (doi:10.1186/s13039-014-0079-2) contains supplementary material, which is available to authorized users.

## Background

Chronic lymphocytic leukemia (CLL) is considered as the most common adult leukemia in Western countries with an estimated incidence of 5.8 in men and of 3.0 in women per 100,000 individuals and per year. It predominantly affects persons with more than 50 years of age [1,2]. A hallmark of CLL is the presence of cytogenetic abnormalities; the latter help to estimate a patient’s prognosis more accurately and also may provide insights into disease pathogenesis [3]. However, banding cytogenetics can only detect aberrations in ~30% of CLL samples [4]. Still, according to molecular (cyto)genetic data the major recurrent aberrations are:

(i) Deletions in 13q14 (50-60% of the samples) associated with a good prognosis, as are deletions in 14q32.33 (12-15% of the samples);
(ii) Trisomy 12 (15-25%) associated with intermediate prognosis; and
(iii) Deletions in 11q22 (*ATM*) (10-20%) or 17p13 (*TP53*) (5-10%) and/or recurrent balanced translocations go together with adverse prognosis [4-9];
(iv) Less frequently observed aberrations in CLL are deletions in 6q associated with intermediate prognosis, 9p21 and 10q23, total or partial trisomies of chromosomes 3, 8, 18, or 19, and duplications in 2p24, the prognostic significance for these aberrations is unknown [1,10,11].

These aberrations were either detected applying cytogenetics and/or interphase fluorescence in situ hybridization (iFISH) [3] or more recently multiplex ligation-dependent probe amplification (MLPA) [7]. While iFISH provides information only for a limited number of genomic targets at the same time [1,5,7] MLPA can detect copy number alterations, methylation pattern changes and/or even point mutations simultaneously in multiple target regions [7,12]. Still iFISH can more reliably detect low level mosaics and mosaics of mono- and biallelic deletions [13].

In this study the efficiency of MLPA was compared with yet in our lab routinely performed cytogenetic and iFISH diagnostics of CLL. Based on the obtained results a new diagnostic scheme is proposed combining MLPA and iFISH leading to a more comprehensive characterization of each individual sample.

## Results

**Figure 1:**
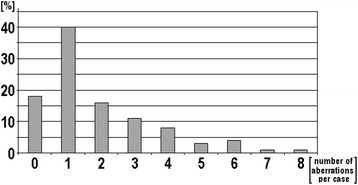
**Number of aberrations present per sample as found in this study after application of all mentioned methods (banding cytogenetics, iFISH and MLPA) – values given in percent.**

85 samples of patients suffering from CLL (Additional file 1: Table S1 and Additional file 2: Table S2) were studied here. Overall, including results from all here applied tests, chromosomal aberrations were detected in 70/85 (~85%) of the studied CLL-samples (Additional file 1: Table S1 and Additional file 2: Table S2). As summarized in Figure 1 between 0 and 8 aberrations were detectable per case. One chromosomal rearrangement per sample could be found most often (40%), followed by no aberration at all and three aberrations per sample. Four or more aberrations per sample were found in less then 10% of the cases.

Overall, 163 aberrations were detected in the 85 studied samples (Table 1, Additional file 2: Table S2). Cytogenetics revealed aberrant karyotypes in 15 (~22%) of the 67 samples where corresponding analyses was successful (Additional file 1: Table S1). In parts the cytogenetic findings could be substantiated by iFISH and or MLPA. As no corresponding probes were included neither in routine iFISH nor in MLPA, 15 (~9%) of the 163 detected aberrations were found additionally by cytogenetics (Table 2). Interestingly, in sample 57 which presented with 5 chromosomal aberrations after banding cytogenetics no aberrations could be detected at all by iFISH or by MLPA. Other samples gave either no, a normal cytogenetic result or a result which also was confirmed by MLPA and/or iFISH (Additional file 1: Table S1).

**Table 1:** Summary of 99 aberrations as detected by MLPA and 146 ones as detected or confirmed by iFISH; samples contributing to the discordant results of MLPA and iFISH are marked with asterisk *, ** or ‘plus-sign’ ^**+**^

**Affected regions**	**Genes**	**Detected in MLPA**	**Detected in iFISH**
amp(2)(p24.3p24.3)	*MYCN*	3	3
amp(2)(p23.2 ~ 23.1p23.2 ~ 23.1)	*ALK*	3	3
del(6)(q21q21)	*FYN*	1	1
del(6)(q23.3q23.3)	*MYB*	2	2
del(6)(q25.1q25.1)	*ESR1*	1	1
del(6)(q27q27)	*SMOC2*	1	1
amp(6)(q27q27)	*SMOC2*	1^+^	0
amp(8)(q24.21q24.21)	*MYC*	1	1
t(9;22)(q34;q11)	*BCR* and *ABL*	n.a.	1
del(11)(q22.3q22.3)	*ATM*	12	14*
+12	*ETV6, CCND2,*	4	6*
	*MDM2*		
del(13)(q14.2q14.2)	*RB1*	10	11**
del(13)(q14.2q14.2)x2	*RB1*	1	10**
del(13)(q14.3q14.3)	*DLEU1, DLEU2,*	35	46*^/^**
	*MIR15A*		
del(13)(q14.3q14.3)x2	*DLEU1, DLEU2,*	7	14**
	*MIR15A*		
del(14)(q32q32)	*IGH*	n.a.	13
rea(14)(q32.33) - > t(14;?)	*IGH*	n.a.	2
rea(14)(q32.33) - > ? + 14	*IGH*	n.a.	1
del(17)(p13.1p13.1)	*TP53*	9	10*
amp(17)(q25.1q25.2)	*UNC13D*	2	2
amp(18)(p11.21q11.21)	*DCC*	2^+^	1
amp(18)(q21.2q21.2)	*RNMT*	2^+^	1
amp(21)(q22.12q22.12)	*RUNX1*	2	2

Those with * are detailed in Table 2, those with ** in Table 4. Those with ^+^ could either not be tested in iFISH due to lack of corresponding probe or, in the two of the tested samples MLPA could not be confirmed by iFISH (routine and confirmatory together), most likely due to too large FISH-probe size.

**Table 2:** **Aberrations only detected by banding cytogenetics in 9 samples of the present study**

**Sample number**	**Aberration only visible in GTG-banding [%]**
1	del(5)(p1?3)[33]
32	-Y[44]
34	-Y[50]
38	t(3;?)(p21;?)[43]
41	-Y[80]
57	der(1)t(1;4)(q1?2;q?31)[90]
	der(4)t(4;?10)(q?31;q24)[90]
	?der(10)t(10;16)(q24;p?11.2)[90]
	der(15)t(1;15)(q1?2;q1?2)[90]
	der(16)t(15;16)(q1?2;p?11.2)[90]
58	der(2)t(2;13)(q?37;q?14)[21]
	?del(6)(p?23)[21]
61	t(3;?)(q2?9;?)[22]
	−7[22]
70	?add(1q)(q4)[50]

Concerning the detection rates, the applied MLPA test found ~58% and routine iFISH ~61% of the 163 aberrations (Table 1, Additional file 2: Table S2). del(13)(q14.3q14.3) was most frequently found, i.e. in ~28% of the samples), followed by del(11)(q22.3q22.3) in ~9%, del(14)(q32q32) in ~8%, and del(13)(q14.2q14.2) and del(17)(p13.1p13.1) in ~6% of the samples, each.

Discordant results of MLPA and routine iFISH were in parts due to the different target regions covered by the tests; thus e.g. del(14)(q32q32) were only detectable by routine iFISH. On the other hand, MLPA detected additional aberrations in 22 samples, three of the patients being without any aberrant findings according to routine iFISH (Additional file 2: Table S2, cases 68–70).

In Table 3 thirteen samples are listed, which had low level mosaic aberrations based on routine iFISH and were not picked up by MLPA. In contrary in Table 4 twelve other samples with similar low level mosaics are listed, which were picked up by MLPA.

**Table 3:** Detailed results in samples contributing to the discordant results of MLPA and iFISH marked with asterisk * in Table 1

**Affected regions**	**Genes**	**Sample number**	**iFISH mosaic [%]**
del(11)(q22.3q22.3)	*ATM*	1	30
del(11)(q22.3q22.3)	*ATM*	2	33
+12	*ETV6*, *CCND2*, *MDM2*	3	15
+12	*ETV6*, *CCND2*, *MDM2*	4	31
del(13)(q14.3q14.3)	*DLEU1*, *DLEU2*, *MIR15A*	5	18
del(13)(q14.3q14.3)	*DLEU1*, *DLEU2*, *MIR15A*	6	10
del(13)(q14.3q14.3)	*DLEU1*, *DLEU2*, *MIR15A*	7	10.5
del(13)(q14.3q14.3)	*DLEU1*, *DLEU2*, *MIR15A*	8	12
del(13)(q14.3q14.3)	*DLEU1*, *DLEU2*, *MIR15A*	9	18.5
del(13)(q14.3q14.3)	*DLEU1*, *DLEU2*, *MIR15A*	10	25
del(13)(q14.3q14.3)	*DLEU1*, *DLEU2*, *MIR15A*	11	34
del(13)(q14.3q14.3)	*DLEU1*, *DLEU2*, *MIR15A*	12	34
del(17)(p13.1p13.1)	*TP53*	13	11.5

**Table 4:** **Detailed results in samples with concordance of MLPA and routine iFISH results but mosaic rates below 40% according to iFISH**

**Affected regions**	**Genes**	**Sample number**	**iFISH mosaic [%]**
del(11)(q22.3q22.3)	*ATM*	14	23.5
del(11)(q22.3q22.3)	*ATM*	15	24
del(11)(q22.3q22.3)	*ATM*	16	11
del(13)(q14.3q14.3)	*DLEU1*, *DLEU2*, *MIR15A*	1	30
del(13)(q14.3q14.3)	*DLEU1*, *DLEU2*, *MIR15A*	2	18
del(13)(q14.3q14.3)	*DLEU1*, *DLEU2*, *MIR15A*	4	20
del(13)(q14.3q14.3)	*DLEU1*, *DLEU2*, *MIR15A*	14	34
del(13)(q14.3q14.3)	*DLEU1*, *DLEU2*, *MIR15A*	17	20
del(17)(p13.1p13.1)	*TP53*	1	16
del(17)(p13.1p13.1)	*TP53*	12	21
del(17)(p13.1p13.1)	*TP53*	18	19
del(17)(p13.1p13.1)	*TP53*	19	36

Table 5 highlights 19 samples which were detected as carrying deletions in 13q14.2 and/or 13q14.3 according to MLPA and iFISH. Still iFISH revealed that there was a mix of monoallelic and biallelic deletion or only biallelic deletion, which could not always be detected by MLPA (Additional file 2: Table S2). Only such cases which had 100% biallelic deletions could be identified undoubtedly (e.g. sample 30); others showed biallelic deletions in MLPA but were indeed a mix of mono- and biallelic ones.

**Table 5:** **Combination of biallelic and/or monoallelic deletion del(13)(q14.2q14.2) and del(13)(q14.3q14.3) – which is not clearly resolved by MLPA**

**Sample number**	**iFISH mosaic [%] del(13)(q14.2q14.2)**		**iFISH mosaic [%] del(13)(q14.3q14.3)**	
	**Monoallelic deletion**	**Biallelic deletion**	**Monoallelic deletion**	**Biallelic deletion**
**2**	0	0	18	14
**4**	45	0	20	0
**12**	52	38	34	0
**13**	0	0	0	98.5
**20**	0	0	0	94
**21**	50	30	0	91
**22**	0	0	5	75
**23**	0	0	5	81
**24**	36	41	16	71
**25**	66	21	18	77
**26**	0	0	25	65
**27**	34	27	36.5	24
**28**	0	0	81	7
**29**	58	24	86	9
**30**	0	0	0	100
**54**	41	39	97	0
**55**	73	5	85	0
**56**	22	58	12	66
**63**	51	38	90	0

Finally, three copy number alterations found by MLPA could not be substantiated by additional iFISH studies (samples 65–67; Additional file 2: Table S2).

**Figure 2:**
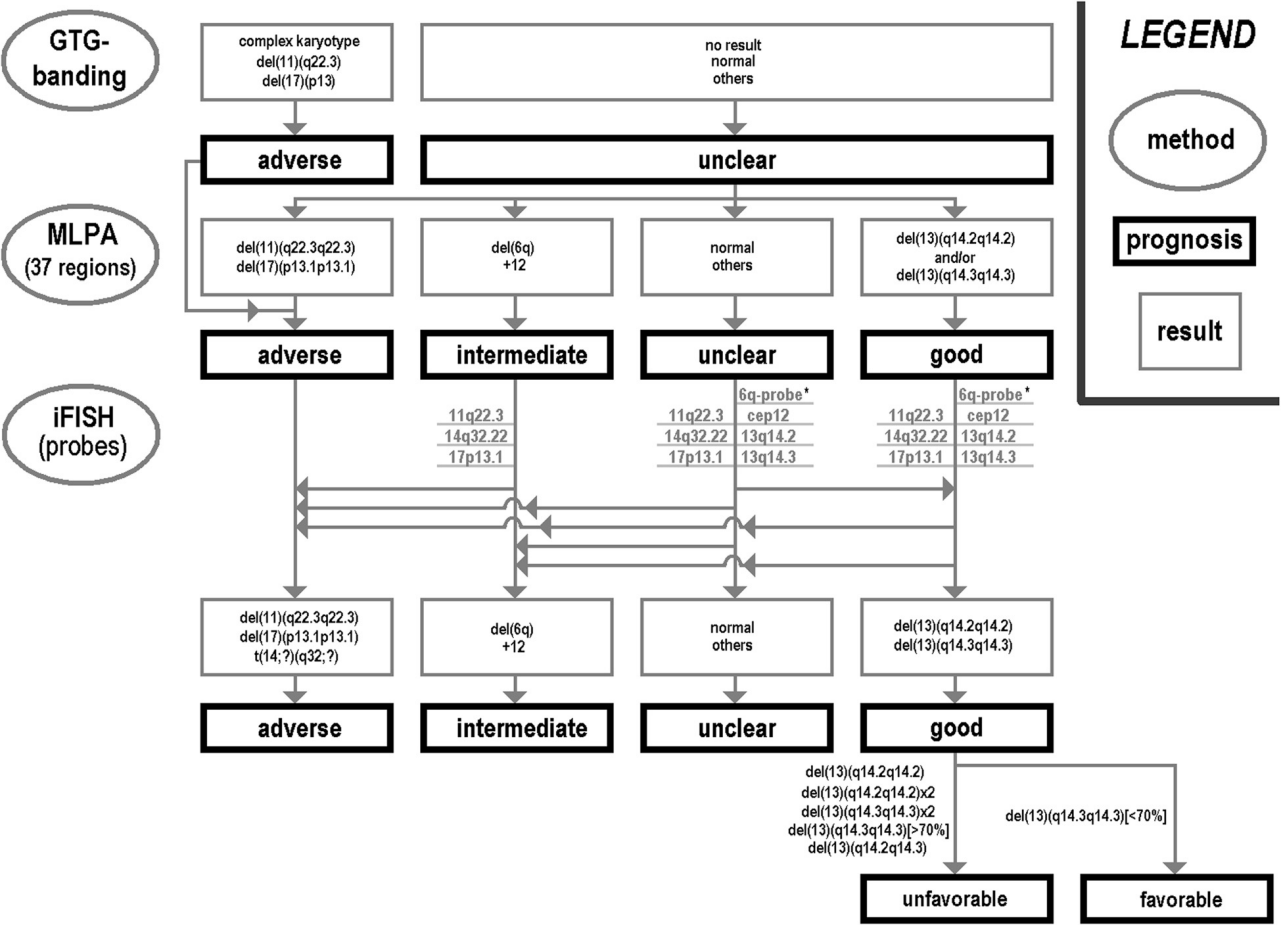
**Suggestion how to proceed when doing MLPA as a primary test after GTG-banding: in case MLPA finds a tumor marker with adverse prognosis no further iFISH analyses is necessary.** In case of an MLPA result suggesting intermediate, unclear or good iFISH for 3 to 6 target regions should be done. A probe for 6q may be also used; however, as case with a del(6q) are rare we would not recommend it at present as really indicated to be applied. According to the obtained results cases need to be regrouped. Finally, iFISH can be used to subclassify cases with good prognosis into such with favorable and unfavorable good prognosis.

**Figure 3:**
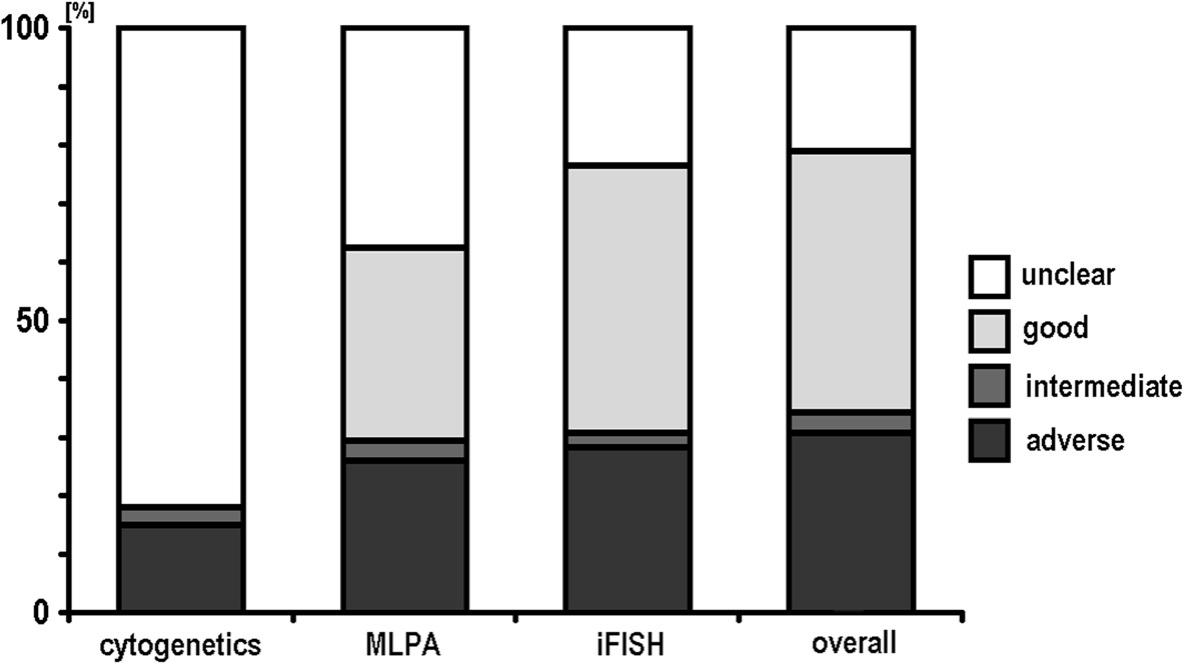
**Detection rates of cytogenetics, MLPA and iFISH as standalone approaches are depicted and compared with overall result combining all three tests as suggested in Figure** 2 **; the corresponding results obtained in the 85 cases were aligned with and are expressed as the resulting prognostic relevance of the identified chromosomal aberrations.**

In Figure 2 a flow is suggested how a CLL-characterization could be performed most comprehensively and straight forward. Figure 3 shows how cases would have been grouped if only cytogenetics, only MLPA or only iFISH would have been done. Tables 6, 7 and 8 highlights how a step by step characterization and corresponding new results of would change the prognosis of the 95 studied cases.

**Table 6:** **Samples from Additional file** 2: **Table S2 are listed according to the groups suggested in Figure** 2

**Results according to MLPA**	**Adverse prognosis**	**Intermediate or unclear prognosis**	**Good prognosis including groups “favorable” and “unfavorable”**	**No aberrations**
**Samples**	1, 10, 12, 14, 15, 16, 17, 18, 19, 24, 34, 35, 38, 39, 54, 58, 61, 63, 64, 65,	5, 37, 62, 68	2*, 4*, 13*, 20*, 21*, 22*, 23*, 25*, 26*, 27*, 28*, 29*, 30*, 40, 41, 42, 43, 44, 45, 46, 47, 48, 49, 50, 51, 52, 53, 55*, 56*, 66, 67, 69	3, 6, 7, 8, 9, 11, 31, 32, 33, 36, 57, 59, 60, 70, 71, 72, 73, 74, 75, 76, 77, 78, 79, 80, 81, 82, 83, 84, 85
**Number of samples per group (absolute)**	***20***	***4***	***32***	***29***
**Number of samples per group (percent)**	***23.5***	***5***	***37.5***	***34***

Samples marked with * have biallelic deletion in 13q14 as substantiated by iFISH or deletion of 13q14.2 and 13q14.3, thus going from favorable to unfavorable subgroup within good prognosis group after iFISH (see Table 7). Figures printed not bold and not in italics are case numbers; figures printed bold and in italics are absolute numbers of samples or same numbers in percent.

**Table 7:** **Regrouping of samples from Table** 6 **after doing additional i-FISH as suggested in Figure** 2

**Results according to MLPA**	**Adverse prognosis**	**Intermediate prognosis**	**Good prognosis “unfavorable”**	**Good prognosis “favorable”**	**No aberrations**
**Samples**	1^+^, 2, 3^+^, 10, 12, 13, 14, 15, 16, 17, 18, 19, 24, 34, 35, 38, 39, 54, 58, 60^+^, 61, 63, 64, 65,	4, 5, 37, 62, 68	20, 21, 22, 23, 25, 26, 27, 28, 29, 30, 43, 44, 45, 46, 47, 48, 49, 50, 51, 52, 53, 55, 56, 66, 67	6, 7, 8, 9, 11, 31, 32, 33, 40, 41, 42, 59, 69	36, 57, 70, 71, 72, 73, 74, 75, 76, 77, 78, 79, 80, 81, 82, 83, 84, 85
**Number of samples per group (absolute)**	***24***	***5***	***25***	***13***	***18***
**Number of samples per group (percent)**	***28***	***6***	***30***	***15***	***21***

Samples marked with ^+^ have rea(14)(q32.33), thus they have to go to the adverse prognosis group. Samples with deletion of 13q14.2 and 13q14.3 detected by MLPA and/or deletion of 13q14.3 in ≥70% of the nuclei detected by iFISH go to unfavorable subgroup within good prognosis group. Figures printed not bold and not in italics are case numbers; figures printed bold and in italics are absolute numbers of samples or same numbers in percent.

**Table 8:** **Final result after including result of GTG-banding based on from Tables** 6 and 7

**Results according to MLPA**	**Adverse prognosis**	**Intermediate or unclear prognosis**	**Good prognosis ** **“unfavorable”**	**Good prognosis “favorable”**	**No aberrations**
**Samples**	1, 2, 3, 10, 12, 13, 14, 15, 16, 17, 18, 19, 24, 34, 35, 38, 39, 54, 57*, 58, 60, 61, 63, 64, 65, 70*	4, 5, 36*, 37, 62, 68	20, 21, 22, 23, 25, 26, 27, 28, 29, 30, 43, 44, 45, 46, 47, 48, 49, 50, 51, 52, 53, 55, 56, 66, 67	6, 7, 8, 9, 11, 31, 32, 33, 40, 41, 42, 59, 69	71, 72, 73, 74, 75, 76, 77, 78, 79, 80, 81, 82, 83, 84, 85
**Number of samples per group (absolute)**	***26***	***6***	***25***	***13***	***15***
**Number of samples per group (percent)**	***31***	***7***	***30***	***15***	***17***

Samples marked with * have additional aberration not detectable by MLPA or routine iFISH. Figures printed not bold and not in italics are case numbers; figures printed bold and in italics are absolute numbers of samples or same numbers in percent.

## Discussion

When diagnostic screening for acquired genetic alteration in hematological malignancies is to be done, banding cytogenetics is still the gold standard, as it enables the untargeted search for gross chromosomal aberrations [14]. Malignant CLL cells derived from bone marrow are known to have a low mitotic index and in many cases only cytogenetically normal cells can be analyzed [4]. Thus, iFISH and MLPA are routinely applied additionally to or even as a replacement in tumorcytogenetics of CLL [7,15].

In this study, after directed diagnostics for 37 genetic loci (MLPA and routine iFISH together), still ~18% of the samples remained without an identified tumor marker. As highlighted by samples 32, 34, 41, 36 (see as well [16]) 38, 57, 58, 61 and 70 this can be due to unusual, not by targeted routine tests covered chromosomal aberrations; besides submicroscopic aberrations like point mutations [2] could be present in those ‘normal’ samples. Interestingly, in over 40% of the studied cases more than only one chromosomal aberration was identified (Figure 1). This may reflect in parts the slow progress of CLL. I.e. the malignancy is detected after acquiring multiple aberrations and not as early as e.g. chronic myelogeneous leukemia (CML), which is already connected with severe clinical signs when only a t(9;22) is found, which is the only aberration in majority of the CML-cases [17].

As mentioned above, MLPA and routine iFISH are targeted tests, both. As they cover in parts different loci it was not unexpected that they have different detection rates. However, one would expect that iFISH technique underestimates the genomic complexity in CLL [1]. Still it is striking that the routine iFISH test found 61% of the 163 aberrations while MLPA only detected 58%, even though routine iFISH applied only 5 probes and MLPA had more than 7 times more, i.e. 37 target regions.

Concerning detection of low level mosaics (10% up to 36% of the cells being aberrant) this study showed that there are about alike amounts of cases being detectable and being missed by MLPA (Tables 3 and 4). There were cases detectable by MLPA with aberrant cell clone sizes down to ~10% according to iFISH (sample 16) and such being not detectable (samples 6, 7, and 8). To the best of our knowledge there are only few previous [18-20] and no systematic studies for the detection rates of low level mosaic in MLPA. Véronèse et al. [7] suggested that all false-negative cases occur in samples with only 12-21% of aberrant cells; thus they considered MLPA detection to be reliable when the fraction of aberrant cells is 25-30%, which is definitely less sensitive than iFISH detection. Overall, this problem has to be kept in mind when doing MLPA exclusively in routine diagnostics.

Still, the findings of this study are in concordance with Stevens-Kroef et al. [21] who claimed an almost perfect correlation between MLPA and iFISH, as long as identical genetic regions are tested in MLPA and iFISH. However, bi- and monoallelic deletions coming together in one sample are not considered in this kind of comparison. Still, all apart from three MLPA findings not detectable in the applied routine iFISH setting could be verified by subsequent targeted iFISH. In the not verified cases this can be due to too small size of the detected copy number alteration, not resolvable by iFISH.

It is well known that there are different clinical prognoses if a del(13)(q14.3q14.3) comes mono- or biallelic and alone or together with a del(13)(q14.2q14.2): larger deletions like del(13)(q14.2q14.3) and biallelic deletions have shorter time to first treatment [1,22,23]. To get reliable information for this question a combination of MLPA and FISH is necessary.

According to Campregher and Hamerschlak [2] the detected aberrations can be grouped in such with adverse, intermediate, good prognosis. Those cases with good prognoses are further subdivided in such cases with favorable and such with less favorable outcome. Especially cases with adverse prognosis have influence on the therapeutic decisions. Taken together with the results of this study we suggest a diagnostic flow as shown in Figure 2.

As both MLPA and routine iFISH have in principle comparable detection rates in CLL, MLPA is more cost efficient than iFISH and it covers a more broad spectrum of target genes [12], we recommend MLPA to be the initial diagnostic test. The impact for the patient carrying rare mutations can be evident: Fabris et al. [11] reported that 2p gain can be present already in early stages of the disease, particularly in those cases characterized by other poor prognostic markers (samples 5, 16 and 63); del(6q) is generally considered as an intermediate-risk factor [1,10] (samples 5 and 68); finally, López et al. [24] reported more rapid disease progression if trisomy 12 is accompanied by additional aberrations rather than if it is the only genetic abnormality (sample 62). Also new data may be acquired, as e.g. the impact of gain of MYC [1] (sample 16) or RUNX1 gene [25] (samples 5 and 69) are still unclear in CLL. If the diagnostic scheme suggested in Figure 2 would have been applied in the 85 patients presented here in 20 of them (23.5%) no iFISH would have been necessary. In those 20 patients (Tables 6, 7 and 8) MLPA would have already identified one or more adverse chromosomal aberrations leading to a therapeutic consequence.

Four patients (Tables 6, 7 and 8) would have been grouped into ‘intermediate prognosis’ after MLPA, one of them just having a trisomy 12 (sample 37). So in this group of patients, only three probes for the adverse prognosis regions should be applied in iFISH testing.

Normal MLPA result as found in 29 samples (= ~34%) all six (or seven, see legend of Figure 2) FISH probes as listed in Figure 2 should be applied to rule out low level mosaics of del(11)(q22.3q22.3), +12, del(13)(q14), del(17)(p13.1p13.1) or del(14)(q32q32). In the present cohort e.g. samples 2 and 13 go to “good prognosis”, samples 3 and 60 to “adverse prognosis” and sample 4 to “intermediate prognosis” group (Tables 6, 7 and 8).

Finally, 32 patients (Tables 6, 7 and 8) have been classified as ‘good prognosis’ after MLPA. Here, the same FISH probes as for normal MLPA result should be used for further subclassification (Figure 2). Again patients then may have to be moved to other prognostic groups if additional or low level mosaics are identified. Also it is known that CLL cases with del(13)(q14.2q14.2) go into unfavorable subgroup, as do such cases with biallelic deletions in 13q14. Finally, del(13)(q14.3q14.3) detected in ≥70% of the cells are also an indication to group a patient in unfavorable subgroup of ‘good prognosis’ group [1,22]. Thus, further I-FISH studies are necessary also for patients with del(13)(q14.2q14.2) and/or del(13)(q14.3q14.3) in MLPA.

In case only MLPA and iFISH would have been done in the presently studied 85 patient still 3 samples would have been misclassified. Thus we suggest in Figure 2 still GTG-banding as the initial test for CLL diagnostics. Compared to a flow just applying banding cytogenetics and routine iFISH for diagnostics of CLL the introduction of the flow from Figure 2 would apply only 344 instead of 425 FISH-probes, i.e. 20% less.

## Conclusion

The present study shows the importance of combining cytogenetics, molecular genetics and molecular cytogenetics to achieve a comprehensive characterization of acquired genetic alterations being present in CLL.

## Methods

### Patients and sample preparation

The present study included 85 samples of patients suffering from CLL (Additional file 1: Table S1 and Additional file 2: Table S2) diagnosed according to standard criteria [26]. The samples were obtained under informed consent of the corresponding patients and according to institutional ethical committee guidelines (Ethical commitee of the Friedrich Schiller University Jena).

DNA from lymphocytes was extracted by a commercial kit (Qiagen, Hilden, Germany) and was derived from different sources: 2 samples from heparinized bone marrow, 8 samples from heparinized blood, and 75 samples from cytogenetically prepared cells fixed in methanol/acetic acid (3:1) – 48 of them derived from bone marrow and 27 from blood (Additional file 1: Table S1).

### GTG-banding and FISH analysis

The blood or bone marrow samples were stimulated with phorbol ester, i.e. 12-O-tetradecanoylphorbol-13-acetate (TPA) and cultivated for 96 hours, and a standard cytogenetic cell preparation following air drying method was done [27]. GTG-banding and iFISH analyses were routinely done in each sample following standard procedures [27,28]. In 67 samples chromosomes could be obtained from the material prepared.

For routine iFISH the following commercially available probe sets (Abbott/Vysis, Wiesbaden, Germany) were used: LSI p53/LSI ATM (in 17p13.1 and 11q22.3), LSI D13S319/LSI 13q34/CEP 12 (in 13q14.3, 13q34 and 12p11.1-q11.1), and LSI IGH dual color, break-apart probe (in 14q32.33).

Additionally, the following probes were used to validate and possibly confirm the results of MLPA:

- from Abbott/Vysis (Wiesbaden, Germany): LSI 13 (RB1 in 13q14.2), CEP 6 (D6Z1 in 6p11.1-q11,1), CEP 17 (D17Z1 in 17p11.1-q11.1) and CEP 18 (D18Z1 in 18p11.1-q11.1);
- from Zytovision (Bremerhaven, Germany): ZytoLight ®SPEC ALK Dual Color Break Apart (in 2p22.32 ~ 22.31), ZytoLight ®SPEC NMYC/2q11 Dual Color (in 2q24.3 and 2q11), ZytoLight ®SPEC MYB Dual Color Break Apart (in 6q23.3), ZytoLight ®SPEC ESR1/CEN 6 Dual Color (in 6q25.1 and 6p11.1-q11.1), ZytoLight ®SPEC CMYC/CEN 8 Dual Color (8q24.21 and 8p11.1-q11.1), ZytoLight ®SPEC ETV6/RUNX1 Dual Color Dual Fusion (in 12p13.2 and 21q22.12); and
- BACPAC Resources Center (Oakland, USA): RP1-142 L7 in 6q21 (gene *FYN*), RP11-318A15 in 17q25.1 (gene *UNC13D*), RP11-346H17 in 18q21.2 (gene *DCC*), RP11-37D8 in 6q27 (gene *SMOC2*) and RP11-411B in 18p11.22 (gene *RNMT*).

For each iFISH analysis, at least 100–200 interphase nuclei were examined per sample and FISH-probe.

### MLPA analysis

MLPA was performed using SALSA MLPA probemix P377-A1 for Hematological Malignancies Kit from (MRC-Holland, Amsterdam, The Netherlands). The P377-A1 probemix kit contains probes for 37 genes covered by overall 52 probes, which have diagnostic or prognostic significant role in hematologic malignancies (see Table 9). MLPA was performed according to the manufacturer’s protocol, which includes three reaction phases: hybridization, ligation, and PCR. Finally, a capillary electrophoresis was used to separate and analyze MLPA PCR products. Genemarker software was used to analyze the peak areas of the MLPA PCR products, and the ratio was normalized to a healthy control. Threshold of detection was set at 0.65-1.35, to minimize the false positive cases.

**Table 9:** **Loci addressed in the commercially available MLPA kit used in this study**

**Targets**	**Loci**	**Number of probes included in kit**
*MYCN*	2p24.3	2
*ALK*	2p23.2 ~ 23.1	1
*MIR145*	5q33.1	1
*EBF1*	5q33.3	2
*MIR146A*	5q33.3	1
*FYN*	6q21	1
*MYB*	6q23.3	1
*ESR1*	6q25.1	1
*SMOC2*	6q27	1
*IKZF1*	7p12.2	3
*CDK6*	7q21.2	1
*RELN*	7q22.1	1
*MET*	7q31.2	1
*DPP6*	7q36.2	1
*MYC*	8q24.21	2
*MTAP*	9p21.3	1
*CDKN2A*	9p21.3	1
*CDKN2B*	9p21.3	1
*PAX5*	9p13.2	2
*PTEN*	9p13.1	1
*PTEN*	10q23.31	1
*ATM*	11q22.3	4
*ETV6*	12p13.2	2
*MDM2*	12q15	1
*CCND2*	12p13.32	1
*RB1*	13q14.2	2
*MIR15A*	13q14.3	1
*DLEU1*	13q14.3	1
*DLEU2*	13q14.3	1
*TP53*	17p13.1	4
*UNC13D*	17q25.1	1
*IKZF3*	17q12	1
*DCC*	18q21.2	1
*RNMT*	18q21.2	1
*CACNA1A*	19p13.13	1
*CHMP2A*	19q13.43	1
*RUNX1*	21q22.12	2

## Additional files

Additional file 1: Table S1. Gender, age and cytogenetic results of the studied cases/samples. Additional file 2: Table S2. Aberrations detected in 85 CLL samples and by which method the corresponding aberrations could be detected.
