# Supplementary material for: Comprehensive chronic lymphocytic leukemia diagnostics by combined multiplex ligation dependent probe amplification (MLPA) and interphase fluorescence in situ hybridization (iFISH)
Source: Mol Cytogenet. 2014 Nov 19;7:79. doi: 10.1186/s13039-014-0079-2 (PMC4247644; doi:10.1186/s13039-014-0079-2)
Supplement: Additional file 1: Table S1. — Gender, age and cytogenetic results of the studied cases/samples. [file 13039_2014_79_MOESM1_ESM.doc]

**Supplementary Table 1**

Gender, age and cytogenetic results of the studied cases/ samples.

Abbreviations: b = cell pellet in Carnoys fixative from blood; B = native blood; bm = cell pellet in Carnoys fixative from bone marrow; BM = native bone marrow; F = female; M = male;

| **case / sample number** | **gender** | **Age [y]** | **DNA extracted from** | **Cytogenetics** |
| --- | --- | --- | --- | --- |
| **1** | M | 83 | bm | 47,XY,-11,+12,+mar[cp3]/  47,XY,del(5)(p1?3),-11,+12,-17,+mar1,+mar2[cp6]/  46,XY[9] |
| **2** | M | 68 | bm | 46,XY |
| **3** | F | 62 | B | n.a. |
| **4** | M | 72 | b | 47,XY,?t(2;14),+12[3]/ 45,X,-Y[4]/46,XY[11] |
| **5** | M | 65 | b | 46,XY |
| **6** | M | 71 | bm | 46,XY |
| **7** | M | 50 | bm | 46,XY |
| **8** | F | 64 | bm | 46,XX |
| **9** | F | 55 | bm | 46,XX |
| **10** | F | 39 | bm | 43~46,XY,del(11)(q2?2q2?4)[cp5]/ 45~46,XY,del(11)(q2?2q2?4),del(15)(q1?1q2?3)[cp11]/ 46,XY[1] |
| **11** | F | 66 | B | 46,XX |
| **12** | F | 50 | b | n.a. |
| **13** | F | 90 | b | n.a. |
| **14** | M | 64 | bm | 46,XY |
| **15** | M | 43 | bm | 46,XY |
| **16** | M | 73 | bm | 46~47,XY,del(11)(q22q2?3),add(17)t(17;?)(p11.2;?)[cp5]/ 45~46,XY,del(11)(q22q2?3),del(17)(p11.2)[cp4]/ 43~46,XY,del(11)(q22q2?3)[cp2]/  46,XY[7] |
| **17** | M | 72 | bm | 46,XY |
| **18** | F | 66 | b | n.a. |
| **19** | F | 71 | b | 46,XX |
| **20** | F | 74 | b | 46,XX |
| **21** | M | 71 | bm | 46,XY |
| **22** | F | 76 | b | 46,XX |
| **23** | M | 62 | b | 46,XY |
| **24** | M | 67 | bm | 46,XY |
| **25** | M | 83 | b | n.a. |
| **26** | M | 79 | B | n.a. |
| **27** | F | 73 | bm | 46,XX |
| **28** | F | 49 | B | 46,XX |
| **29** | F | 69 | b | n.a. |
| **30** | M | 78 | b | 46,XY |
| **31** | M | 74 | bm | 46,XY |
| **32** | M | 71 | b | 45,X,-Y[cp8]/ 46,XY[10] |
| **33** | F | 63 | bm | 46,XX |
| **34** | M | 77 | bm | 46,XY,del(11)(q?21),add(20)(p13)[7]/  45,X,-Y[10]/  46,XY[3] |
| **35** | M | 53 | bm | 46,XY |
| **36** | M | 73 | bm | 45,X,-Y,t(9;22)(q34;q11) |

**Supplementary Table 1 (ctd.)**

| **case / sample number** | **gender** | **age [y]** | **DNA extracted from** | **Cytogenetics** |
| --- | --- | --- | --- | --- |
| **37** | M | 74 | bm | 45,X,-Y[2]/  47,XY,+12[1]/  48,XY,-6,-8,+12,+mar,+mar,+mar[1]/  46,XY[14] |
| **38** | M | 65 | bm | 46,XY,?t(3;?)(p21;?),add(17)(p?12) or t(17;?),-18,+mar[cp7]/  46,XY[9] |
| **39** | F | 74 | B | 46,XX |
| **40** | F | 72 | b | 46,XX |
| **41** | M | 72 | bm | 45,X,-Y[4]/  46,XY[16] |
| **42** | M | 51 | bm | 46,XY |
| **43** | F | 48 | b | 46,XX |
| **44** | F | 47 | b | 46,XX |
| **45** | F | 79 | bm | 46,XX |
| **46** | M | 67 | bm | n.a. |
| **47** | M | 67 | bm | n.a. |
| **48** | M | 68 | bm | 46,XY |
| **49** | M | 61 | b | 46,XY |
| **50** | M | 73 | bm | 46,XY |
| **51** | M | 75 | bm | 46,XY |
| **52** | M | 77 | b | n.a. |
| **53** | M | 54 | b | 46,XY |
| **54** | M | 59 | bm | n.a. |
| **55** | M | 59 | b | 46,XY |
| **56** | F | 47 | B | 46,XX |
| **57** | M | 71 | bm | 46,XY,der(1)(t(1;4)(q1?2;q?31),der(4)t(4;?10)(q?31;q24),  ?der(10)t(10;16)(q24;p?11.2),der(15)t(1;15)(q1?2;q1?2),  der(16)t(15;16)(q1?2;p?11.2)[17]/  46,XY[2] |
| **58** | M | 73 | bm | 45,XY,der(2)t(2;13)(q?37;q?14),?del(6)(p?23),del(11)(q?21),  der(12)t(12;13)(q?24;q?22),-13[cp4]/46,XY[19] |
| **59** | M | 72 | bm | 46,XY |
| **60** | M | 54 | B | n.a. |
| **61** | F | 74 | bm | 46,XX,i(17)(q10)[1]/  46,XX,+12,i(17)(q10),-21[9]/  46,XX,t(3;?)(q2?9;?)[4],-7[4],+12[4],i(17)(q10)[4][cp4]/  46,XX[4] |
| **62** | F | 65 | bm | 46,XX |
| **63** | M | 72 | b | n.a. |
| **64** | F | 73 | bm | 46,XX,add(11)(q?22)[3]/  46,XX[12] |
| **65** | M | 54 | b | 46,XY |
| **66** | F | 69 | bm | 46,XX |
| **67** | M | 53 | bm | 46,XY |
| **68** | M | 53 | b | 46,XY |
| **69** | M | 75 | bm | 46,XY |
| **70** | M | 56 | b | 46,XY,?add(1q)(q4)[3]/  46,XY[3] |
| **71** | F | 58 | BM | n.a. |
| **72** | F | 73 | B | n.a. |
| **73** | M | 66 | bm | 46,XY |

**Supplementary Table 1 (ctd.)**

| **case / sample number** | **gender** | **age [y]** | **DNA extracted from** | **Cytogenetics** |
| --- | --- | --- | --- | --- |
| **74** | M | 74 | bm | 46,XY |
| **75** | F | 51 | bm | 46,XX |
| **76** | M | 52 | bm | 46,XY |
| **77** | M | 63 | bm | n.a. |
| **78** | M | 60 | b | 46,XY |
| **79** | M | 72 | b | n.a. |
| **80** | F | 49 | bm | 46,XX |
| **81** | F | 64 | bm | 46,XX |
| **82** | F | 72 | bm | 46,XX |
| **83** | M | 82 | b | 46,XY |
| **84** | M | 74 | BM | 46,XY |
| **85** | F | 72 | bm | n.a. |
