# Supplementary material for: Comprehensive chronic lymphocytic leukemia diagnostics by combined multiplex ligation dependent probe amplification (MLPA) and interphase fluorescence in situ hybridization (iFISH)
Source: Mol Cytogenet. 2014 Nov 19;7:79. doi: 10.1186/s13039-014-0079-2 (PMC4247644; doi:10.1186/s13039-014-0079-2)
Supplement: Additional file 2: Table S2. — Aberrations detected in 85 CLL samples and by which method the corresponding aberrations could be detected. [file 13039_2014_79_MOESM2_ESM.doc]

**Supplementary Table 2**

Aberrations detected in 85 CLL samples and by which method the corresponding aberrations could be detected. Abbreviations: + = detected, (+) = detected but not specific as bi- or monoallelic deletion, o = not tested, - not detected, n = no aberration.

| **sample number** | **aberrations [%]** | **iFISH routine** | **MLPA** | **iFISH confirmatory for MLPA** |
| --- | --- | --- | --- | --- |
| **1** | del(5)(p1?3)[33]  del(11)(q22.3q22.3)[30] +12[70] del(13)(q14.3q14.3)[30] rea(14)(q32.33)[28] -> ?+14 del(17)(p13.1p13.1)[16]  amp(17)(q25.1q25.2)[40] | o  +  +  +  +  +  o | o  -  +  +  o  +  + | o  o  o  o  o  o  + |
| **2** | del(11)(q22.3q22.3)[33] del(13)(q14.3q14.3)[18] del(13)(q14.3q14.3)x2[14] | +  +  + | -  +  (+) | o  o  o |
| **3** | +12[15] rea(14)(q32.33)[52] -> t(14;18)(q32;q21) | +  + | -  o | o  o |
| **4** | +12[31] del(13)(q14.2q14.2)[45] del(13)(q14.3q14.3)[20] | +  o  + | -  +  + | o  +  o |
| **5** | amp(2)(p24.3p24.3)[60] amp(2)(p23.2~23.1p23.2~23.1)[63] del(6)(q23.3q23.3)[68] del(6)(q25.1q25.1)[65] del(6)(q27q27)[23] del(13)(q14.3q14.3)[18] del(14)(q32q32)[65]  amp(21)(q22.12q22.12)[86] | o  o  o  o o + + o | +  +  +  +  +  -  o  + | +  +  +  +  +  o  o  + |
| **6** | del(13)(q14.3q14.3)[10] | + | - | o |
| **7** | del(13)(q14.3q14.3)[10.5] | + | - | o |
| **8** | del(13)(q14.3q14.3)[12] | + | - | o |
| **9** | del(13)(q14.3q14.3)[18.5] | + | - | o |
| **10** | del(11)(q22.3q22.3)[98] del(13)(q14.3q14.3)[25] | +  + | +  - | o o |
| **11** | del(13)(q14.3q14.3)[34] | + | - | o |
| **12** | del(13)(q14.2q14.2)[52] del(13)(q14.2q14.2)x2[38] del(13)(q14.3q14.3)[34] del(17)(p13.1p13.1)[21] | o o +  + | + (+)  -  + | +  +  o o |
| **13** | del(13)(q14.3q14.3)x2[98.5] del(17)(p13.1p13.1)[11.5] | +  + | + - | o  o |
| **14** | del(11)(q22.3q22.3)[23.5] del(13)(q14.3q14.3)[34] | +  + | +  + | o  o |
| **15** | del(11)(q22.3q22.3)[24] | + | + | o |
| **16** | amp(8)(q24.21q24.21)[21] del(11)(q22.3q22.3)[11] del(17)(p13.1p13.1)[86] | o  +  + | +  +  + | +  o  o |
| **17** | del(13)(q14.3q14.3)[20] del(17)(p13.1p13.1)[40] | +  + | +  + | o  o |
| **18** | del(13)(q14.3q14.3)[90.5] del(17)(p13.1p13.1)[19] | +  + | +  + | o  o |
| **19** | del(17)(p13.1p13.1)[36] | + | + | o |
| **sample number** | **aberrations [%]** | **iFISH routine** | **MLPA** | **iFISH confirmatory for MLPA** |
| **20** | del(13)(q14.3q14.3)x2[94] del(14)(q32q32)[97] | +  + | + o | o  o |
| **21** | del(13)(q14.2q14.2)[50] del(13)(q14.2q14.2)x2[30] del(13)(q14.3q14.3)x2[91] | o  o  + | +  (+) + | +  +  o |
| **22** | del(13)(q14.3q14.3)[5] del(13)(q14.3q14.3)x2[75] | +  + | (+)  + | o  o |
| **23** | del(13)(q14.3q14.3)[5] del(13)(q14.3q14.3)x2[81] | +  + | (+)  + | o  o |
| **24** | del(11)(q22.3q22.3)[88] del(13)(q14.2q14.2)[36] del(13)(q14.2q14.2)x2[41] del(13)(q14.3q14.3)[16] del(13)(q14.3q14.3)x2[71] | +  o  o  +  + | +  +  (+) (+)  + | o  +  + o  o |
| **25** | del(13)(q14.2q14.2)[66]  del(13)(q14.2q14.2)x2[21] del(13)(q14.3q14.3)[18] del(13)(q14.3q14.3)x2[77] | o  o  +  + | +  (+)  (+)  + | +  +  o  o |
| **26** | del(13)(q14.3q14.3)[25] del(13)(q14.3q14.3)x2[65] | +  + | +  (+) | o  o |
| **27** | del(13)(q14.2q14.2)[34] del(13)(q14.2q14.2)x2[27] del(13)(q14.3q14.3)[36.5] del(13)(q14.3q14.3)x2[24] del(14)(q32q32)[12] | o  o  +  +  + | +  (+)  +  (+)  o | +  +  o  o  o |
| **28** | del(13)(q14.3q14.3)[81] del(13)(q14.3q14.3)x2[7] | +  + | +  (+) | o  o |
| **29** | del(13)(q14.2q14.2)[58] del(13)(q14.2q14.2)x2[24] del(13)(q14.3q14.3)[86] del(13)(q14.3q14.3)x2[9] | o  o  +  + | +  (+)  +  (+) | +  +  o  o |
| **30** | del(13)(q14.3q14.3)x2[100] | + | + | o |
| **31** | del(14)(q32q32)[92] | + | o | o |
| **32** | del(14)(q32q32)[81]  -Y[44] | +  o | o  o | o  o |
| **33** | del(14)(q32q32)[58] | + | o | o |
| **34** | del(11)(q22.3q22.3)[90] del(14)(q32q32)[90]  -Y[50] | +  +  o | +  o  o | o  o  o |
| **35** | del(11)(q22.3q22.3)[77] | + | + | o |
| **36** | t(9;22)(q34;q11)[94] | + | o | o |
| **37** | +12[49.5] | + | + | o |
| **38** | t(3;?)(p21;?)[43]  del(17)(p13.1p13.1)[89] | o  + | o  + | o  o |
| **39** | del(17)(p13.1p13.1)[77] | + | + | o |
| **40** | del(13)(q14.3q14.3)[52] | + | + | o |
| **41** | del(13)(q14.3q14.3)[60]  -Y[80] | +  o | +  o | o  o |
| **42** | del(13)(q14.3q14.3)[68] | + | + | o |
| **43** | del(13)(q14.3q14.3)[70.5] | + | + | o |
| **sample number** | **aberrations [%]** | **iFISH routine** | **MLPA** | **iFISH confirmatory for MLPA** |
| **44** | del(13)(q14.3q14.3)[73] | + | + | o |
| **45** | del(13)(q14.3q14.3)[73] | + | + | o |
| **46** | del(13)(q14.3q14.3)[80] | + | + | o |
| **47** | del(13)(q14.3q14.3)[80] | + | + | o |
| **48** | del(13)(q14.3q14.3)[81] | + | + | o |
| **49** | del(13)(q14.3q14.3)[83] | + | + | o |
| **50** | del(13)(q14.3q14.3)[85] | + | + | o |
| **51** | del(13)(q14.3q14.3)[91] | + | + | o |
| **52** | del(13)(q14.3q14.3)[94] | + | + | o |
| **53** | del(13)(q14.3q14.3)[94.5] | + | + | o |
| **54** | del(11)(q22.3q22.3)[98]  del(13)(q14.2q14.2)[41]  del(13)(q14.2q14.2)x2[39]  del(13)(q14.3q14.3)[97] | +  o  o  + | +  +  (+)  + | o  +  +  o |
| **55** | del(13)(q14.2q14.2)[73]  del(13)(q14.2q14.2)x2[5]  del(13)(q14.3q14.3)[85] | o  o  + | +  (+)  + | +  +  o |
| **56** | del(13)(q14.2q14.2)[22]  del(13)(q14.2q14.2)x2[58]  del(13)(q14.3q14.3)[12]  del(13)(q14.3q14.3)x2[66] | o  o  +  + | +  (+)  +  (+) | +  +  o  o |
| **57** | der(1)t(1;4)(q1?2;q?31)[90]  der(4)t(4;?10)(q?31;q24)[90]  ?der(10)t(10;16)(q24;p?11.2)[90]  der(15)t(1;15)(q1?2;q1?2)[90]  der(16)t(15;16)(q1?2;p?11.2)[90] | o  o  o  o  o | o  o  o  o  o | o  o  o  o  o |
| **58** | der(2)t(2;13)(q?37;q?14)[21]  ?del(6)(p?23)[21]  del(11)(q22.3q22.3)[87] del(13)(q14.3q14.3)[87] del(14)(q32q32)[85] | o  o  +  +  + | o  o  +  +  o | o  o  o  o  o |
| **59** | del(14)(q32q32)[85] | + | o | o |
| **60** | rea(14)(q32.33)[96] -> t(14;?)(q32;?) | + | o | o |
| **61** | t(3;?)(q2?9;?)[22]  -7[22]  +12[78] del(14)(q32q32)[94]  del(17)(p13.1p13.1)[95]  amp(17)(q25.1q25.2)[22] | o  o  +  +  +  o | o  o  +  o  +  + | o  o  o  o  o  + |
| **62** | +12[80] del(13)(q14.3q14.3)[62]  amp(18)(p11.21q11.21)[75] amp(18)(q21.2q21.2)[75] | +  +  o  o | +  +  +  + | o  o  +  + |
| **63** | amp(2)(p24.3p24.3)[62.5] amp(2)(p23.2~23.1p23.2~23.1)[62.5]  del(11)(q22.3q22.3)[95]  del(13)(q14.2q14.2)[51]  del(13)(q14.2q14.2)x2[38] del(13)(q14.3q14.3)[90] del(14)(q32q32)[91] | o  o  +  o  o  +  + | +  +  +  +  (+)  +  o | +  +  o  +  +  o  o |
| **sample number** | **aberrations [%]** | **iFISH routine** | **MLPA** | **iFISH confirmatory for MLPA** |
| **64** | amp(2)(p24.3p24.3)[65]  amp(2)(p23.2~23.1p23.2~23.1)[75] del(11)(q22.3q22.3)[83] del(13)(q14.3q14.3)[58.5] | o  o  +  + | +  +  +  + | +  +  o  o |
| **65** | amp(6)(q27q27)[?] del(11)(q22.3q22.3)[93] del(13)(q14.3q14.3)[96] | o  +  + | +  +  + | -  o  o |
| **66** | del(13)(q14.3q14.3)[90] del(14)(q32q32)[81]  amp(18)(q21.2q21.2)[?] | +  +  o | +  o  + | o  o  - |
| **67** | del(13)(q14.3q14.3)[60] del(14)(q32q32)[80]  amp(18)(p11.21q11.21)[?] | +  +  o | +  o  + | o  o  - |
| **68** | del(6)(q21q21)[33] del(6)(q23.3q23.3)[92] | o  o | +  + | +  + |
| **69** | amp(21)(q22.12q22.12)[50] | o | + | + |
| **70** | ?add(1q)(q4)[50] | o | o | o |
| **71** | None | n | n | o |
| **72** | None | n | n | o |
| **73** | None | n | n | o |
| **74** | none | n | n | o |
| **75** | none | n | n | o |
| **76** | none | n | n | o |
| **77** | none | n | n | o |
| **78** | none | n | n | o |
| **79** | none | n | n | o |
| **80** | none | n | n | o |
| **81** | none | n | n | o |
| **82** | none | n | n | o |
| **83** | none | n | n | o |
| **84** | none | n | n | o |
| **85** | none | n | n | o |
